# A compilation of 13 patients with metastatic colorectal cancer and concomitant *BRAF* and *RAS* family mutations

**DOI:** 10.3389/fonc.2025.1621412

**Published:** 2025-08-25

**Authors:** Jigisha Srivastav, Morgan E. Lehman, Joni K. Evans, Ravi Paluri, Caio Max Sao Pedro Rocha Lima

**Affiliations:** ^1^ Department of Internal Medicine, Wake Forest University School of Medicine, Winston-Salem, NC, United States; ^2^ Department of Biostatistics and Data Science, Wake Forest University School of Medicine, Winston-Salem, NC, United States; ^3^ Department of Hematology and Oncology, Wake Forest University School of Medicine, Winston-Salem, NC, United States

**Keywords:** colorectal cancer, BRAF mutation, KRAS mutation, NRAS mutation, RAS family mutation, concurrent RAS/BRAF variants, clinical-pathological features

## Abstract

**Introduction:**

Metastatic colorectal cancer (mCRC) exhibits significant heterogeneity in molecular profiles, influencing treatment response and patient outcomes. Mutations in v-raf murine sarcoma viral oncogene homolog B1 (*BRAF*) and rat sarcoma (*RAS*) family genes are commonly observed in mCRC. Though originally thought to be mutually exclusive, recent data have shown that patients may present with concomitant *RAS* and *BRAF* mutations, posing unique challenges and implications for clinical management.

**Methods:**

Below we present a retrospective study on 13 patients with concomitant *BRAF* and *RAS* (*KRAS, NRAS*) mutations in mCRC and describe their clinical features and treatment outcomes. We reviewed over 750 samples from a database of CRC patients from Guardant360 and FoundationOne kept by the Wake Forest Baptist Health Comprehensive Cancer Center. The study population included patients greater than the age of 18 who were diagnosed with mCRC harboring both *BRAF* and *RAS* mutations, as identified by next generation sequencing.

**Results:**

Thirteen mCRC patients, 61.5% male, with a median age of diagnosis of 64.4 years had concomitant *BRAF* and *RAS* mutation. 61.5% of patients had right-sided primary disease. 61.5% patients had mutations in codon 12 of *KRAS*, 15.4% had *BRAF* G466V, and 15.4% had *BRAF* V600E mutations. 69.2% patients had liver metastasis, 23.1% had peritoneal metastases and 7.7% suffered metastasis to supraclavicular, retroperitoneal, and mesenteric lymph nodes. Median time from diagnosis of stage IV disease to progression was 25.3 months and median overall survival was 4.9 years.

**Discussion:**

This study adds more insight to the limited existing data regarding rare mCRC cases with concomitant *BRAF* and *RAS* family mutations and exposes the need for future research on larger populations of this rare subset of patients.

## Introduction

Colorectal cancer (CRC) is the third most common cause of cancer-related death in both men and women and ranks second in cancer-related deaths overall (1). Approximately 65% of patients diagnosed with CRC will either present with metastatic cancer at diagnosis or develop distant metastasis later in the course of the disease. The treatment of metastatic CRC (mCRC) has been individualized due to biomarkers that can be both prognostic and predictive, aiding in treatment decisions (2, 3).

Rat sarcoma (*RAS*) family genes (*KRAS, NRAS*) and v-raf murine sarcoma viral oncogene homolog B1 (*BRAF*) play a key role in the epithelial growth factor receptor (EGFR) pathway, an essential pathway for survival of cancer cells. Mutations in *RAS* family and *BRAF* genes lead to loss of cell cycle regulation and are the primary driver mutations for colorectal carcinogenesis (4). Specifically, *RAS* mutations are activating mutations which increase the expression and activity of RAS, driving an increase in cellular mitosis resulting in continuous proliferation and cell growth leading to carcinogenesis (5, 6). *RAS* mutations are present in about 40% of mCRC cases (5). *KRAS* is the most common of the *RAS* family genes to be involved in CRC and majority have missense gain of function mutation including codon 12, 13, and 61, most commonly G12D, G12V, G13D. Other codons that can be involved are 59, 117, 146. NRAS mutations in CRC are usually found in codons 12, 13, 61, most commonly G12A, G12D, G13S, Q61Q. The presence of a *RAS* mutation has been found to be a negative prognostic factor (5).

Conversely, *BRAF* mutations are only present in about 8-12% of mCRC cases (5). These mutations drive MAP-kinase pathway activation which leads to cellular growth, proliferation, and differentiation (5). *BRAF* mutations have been divided into three categories based upon their RAS dependency, kinase activity, and susceptibility to inhibitors. Class I are RAS-independent, kinase-activating V600 mutations, which signal as monomers and are characteristically responsive to BRAF inhibitors. Class II are RAS-independent, kinase-activating non-V600E mutations, which signal as dimers. Class III are RAS-dependent, BRAF variants characterized by the activation of tyrosine kinase receptors and enhancement of the ERK pathway. Class III mutations are associated with a better overall survival (OS), left-sided tumors, and absence of lymph node and peritoneal metastases (7).

CRC with a *RAS* mutation (8) or class I or II *BRAF* mutations (9) may be resistant to anti-EGFR therapies (5, 10). *BRAF* mutations in mCRC can be targeted with anti-BRAF agents and anti-EGFR agents in combination (11). Moreover, patients with *BRAF* V600E mutations may benefit from first-line therapy with encorafenib, cetuximab, and mFOLFOX 6 based on the BREAKWATER phase III trial (12). Until recently, mutations in *BRAF* and *RAS* were originally considered to be mutually exclusive in CRC and as such, very limited data have been published on their co-occurrence. One study showed that concurrent *KRAS/BRAF* mutations are reported in about 0.05% of metastatic CRC cases (5). We present a retrospective study that examines 13 patients with concomitant *RAS* and *BRAF* mutations in mCRC and its association with clinicopathological features and outcomes.

## Materials and methods

We reviewed 763 samples from Guardant360 and FoundationOne databases kept by the Wake Forest Baptist Health Comprehensive Cancer Center. Descriptions of these databases can be found in **Supplementary Tables 1** and **2**. The study population included patients greater than age 18 with mCRC who were identified to have both a *BRAF* and *RAS* mutation by next generation sequencing (NGS). From these databases, we identified patients that had concomitant *BRAF* and *RAS* mutations in the CRC population. Further, we excluded patients who did not have mCRC and/or who had very limited data on chart review. We also excluded patients who had *ARF* mutations or *RAF* mutations. Ultimately, we identified 13 patients with mCRC and concomitant *BRAF* and *RAS* family gene mutations. NGS data was obtained on all 13 patients. The NGS sample was obtained from primary tumor (colon) in four patients, metastatic site in seven patients, and from circulating tumor DNA (ctDNA) in five patients. Three patients had samples obtained from both metastatic site and ctDNA. A retrospective record review was conducted in January 2024 on this sample to examine specific demographics and clinical features of the select population including sex, age at initial diagnosis and at diagnosis of stage IV disease, location of CRC, stage of the CRC at initial diagnosis, type of *RAS* mutation, type of *BRAF* mutation, location of metastasis, first-line treatment of metastatic cancer, second and further lines of treatment of the patients CRC, response to therapy, duration of response, time to progression, and survival length. We also reviewed tumor mutation burden (TMB) and microsatellite instability (MSI) status for each patient. MSI status was determined through NGS for eight patients. The others were determined through an alternative method. We used primarily descriptive statistics (means or percentages), and estimated time to progression and overall survival using Kaplan-Meier methods.

## Results

Eight patients (61.5%) were newly diagnosed with stage IV disease, the other five were diagnosed at stages I-III and later progressed to stage IV disease (**Table 1**). The following analyses were conducted on patients from the time of stage IV presentation. Patients were between 32 and 92 years old (median age 64.4). Eight (61.5%) were male and five (38.5%) were female. Eight patients had right-sided primary disease, four had left-sided, and one had synchronous primaries of the ascending and sigmoid colon. **Table 2** shows each patient’s unique combination of mutations, the origin of the NGS sample, and the exon of each gene. *KRAS* mutation in codon 12 was the most prevalent *RAS* family mutation. Only two patients with a *RAS* family mutation harbored a *BRAF* V600E mutation.

**Table 1:** Patient demographics and tumor characteristics.

Patient Demographics	
Median Age at Diagnosis of Stage IV Disease​ (IQR)	64.4 (59.3, 68.2)​
Male (%)​	8 (61.5%)​
Female (%)	5 (38.5%)
Tumor Characteristics	
	N (%)
Primary Tumor Location	
Cecum ​	5 (38.5%) ​
Sigmoid Colon ​	3 (23.1%)​
Right Colon​	2 (15.4%)​
Synchronous ascending and sigmoid colon​	1 (7.7%)​
Transverse Colon​	1 (7.7%)​
Rectum ​	1 (7.7%) ​
Clinical Stage at Diagnosis	
I​	1 (7.7%)​
II​	1 (7.7%)​
III​	3 (23.1%) ​
IV ​	8 (61.5%) ​
Metastasis Location	
Liver​	5 (38.5%)​
Liver and Lung​	2 (15.4%)​
Liver and Brain​	1 (7.7%)​
Lung and Brain ​	1 (7.7%)​
Peritoneum/Omentum​	1 (7.7%)​
Peritoneum​	1 (7.7%)​
Liver, Peritoneum, Ovaries​	1 (7.7%)​
Supraclavicular, RP, Mesenteric Lymph Nodes​	1 (7.7%)​

**Table 2:** RAS and BRAF mutations.

Patient ID​	Origin of Sample for NGS (primary, met, or ctDNA)	RAS Mutation​	Exon	BRAF Mutation​	Exon
1​	ctDNA	KRAS Q61H, G12D​	3, 2	BRAF K570T​	13
2​	Met (liver)	KRAS G12D​	2	BRAF K601N​	15
3​	Met (small intestine)	KRAS K182N​	6	BRAF R682W​	16
4​	Primary (colon)	KRAS G12D	2	BRAF G466V​	11
5​	Met (peritoneum)	KRAS G12V​	2	BRAF K578R​	13
6​	Met (liver) and ctDNA	KRAS R1264Q, KRAS E168*​	5	BRAF K205N​	5
7​	Met (bile duct) and ctDNA	KRAS P34R​	2	BRAF G466V​	11
8​	Primary (colon)	KRAS G12S	2	BRAF N581Y​	13
9​	Primary (colon) and ctDNA	KRAS Q61H, G12D, G12R​	3, 2, 2	BRAF E26A​	1
10​	ctDNA	NRAS Q61K​	3	BRAF V600E​	15
11	Met (lung)	KRAS G12V	2	BRAF T241M​	6
12​	Met (lymph node)	NRAS Q61R​	3	BRAF V600E​	15
13​	Primary (colon)	KRAS G12V	2	BRAF Q257R​	6

Efficacy results include a median time to progression (from stage IV diagnosis) of 25.3 months (95% CI 8.3, 39.5). Nine patients (69.2%) were progression-free at 12 months and three patients (27.7%) were progression free at 36 months. Nine patients (69.2%) have died. The median time to death (from diagnosis of stage IV disease) was 4.9 years (95% CI 3.5, 5.5). 71.8% of patients survived four years, 13.7% survived six years. Three patients (23.1%) did not have progression of their disease at 30, 40, and 60 months. Two of these patients possessed *KRAS* G12D mutation. **Table 3** shows additional information regarding the patients who did not have progression, including MSI and TMB status. MSI status is categorized into microsatellite instability high (MSI-H) and microsatellite stable (MSS).

**Table 3:** Characteristics of the patients without progression* and were still living at the time of data collection.

Patient ID	Sex	CRC Location	RAS mutation	BRAF mutation	Pathology	MSI Status	TMB	Date Diagnosed**	Location of Metastasis
2	M	Sigmoid	KRAS G12D	BRAF K601N	Adenocarcinoma	MSS	4 muts/mB	1/21/2022	Liver, Lung
4	F	Cecum	KRAS G12D	BRAF G466V	Adenocarcinoma	MSS	5 muts/mB	6/2020	Liver
12	F	Cecum	NRAS Q61R	BRAF V600E	Adenocarcinoma	MSI-High	39 muts/mB	1/2018	Supraclavicular, retroperitoneal, and mesenteric lymph nodes

*Absence of progression at 30, 40 and 60 months **With metastatic disease.

## Discussion

The presence of concomitant *BRAF* and *RAS* mutations has historically been thought to be mutually exclusive in mCRC. One study showed 0.064% in a population of 6,251 (13). A review of 11 papers identified a total of only 30 cases of concomitant *RAS* and *BRAF* mutations in mCRC (5). The present study describes 13 patients with concomitant *RAS* and *BRAF* mutations in mCRC.

In our cohort of patients, we identified many different combinations of concomitant *BRAF* and *RAS* mutations (**Table 2**). Primary tumor location and sites of metastasis, as listed in **Table 1**, show that the majority of the patients had liver metastasis, 69.2%. Three patients (23.1%) had peritoneal metastases and one (7.7%) with metastasis to supraclavicular, retroperitoneal (RP), and mesenteric lymph nodes. This distribution appears similar to what has been reported with *BRAF*-mutant mCRC patients except for the percentage of RP nodes (14, 15). Our cohort shares some features with *BRAF*-mutant-only mCRC, but the lower incidence of RP node involvement and higher liver-predominant disease suggests that concomitant *RAS* mutation may modify the metastatic pattern.

Individually, *BRAF* and *RAS* mutations in mCRC are associated with adverse prognosis. Prior studies have reported a median overall survival (OS) of 18.9 months in patients with *BRAF*-mutated mCRC, compared to 33.2 months in those with wild-type *BRAF* tumors (16) and median OS of 27.5 months for *RAS* wild-type and 17.3 months for *RAS*-mutated tumors (17). However, the presence of *RAS* and *RAF* mutations may reflect a more heterogeneous biology than previously appreciated since in our cohort, the median OS from the time of diagnosis of metastatic disease is 4.9 years (95% CI 3.5, 5.5) and three patients were still alive at the time of data collection.

Several factors may contribute to this prolonged survival, including access to sequential lines of effective systemic therapy, the use of multimodal treatment approaches, and the possibility of distinct tumor biology within this dual-mutant subset. Furthermore, these outcomes may reflect careful patient selection, as well as improvements in supportive care and molecularly-guided treatment strategies over time. This observation indicates the need to re-examine the prognostic assumptions in molecularly defined subgroups and highlights the importance of continued molecular characterization and individualized treatment planning.

Of the three patients without progression at 30, 40, and 60 months and were still alive at the time of data collection, one had MSI-H disease with a high TMB of 39 mutations per megabase (muts/mB). This patient responded favorably to the immune checkpoint inhibitor pembrolizumab and remained progression-free at the time of data collection. Pembrolizumab is a standard first-line therapy for mCRC in patients with MSI-H or mismatch repair–deficient (dMMR) tumors (18, 19). Emerging data suggest that immunotherapy may be equally effective in MSI-H mCRC regardless of *BRAF* mutation status (20, 21). Ongoing trials, such as SEAMARK, are investigating whether adding targeted therapy offers additional benefit in this population, or whether immunotherapy alone is sufficient (22). Further, the CheckMate 8HW trial showed that patients with MSI-H/dMMR mCRC treated with nivolumab plus ipilimumab had significantly longer progression-free survival (PFS) when compared to treatment with chemotherapy alone, including patients with baseline *RAS* or *BRAF* mutations (23). However, it remains unclear whether this included patients with co-occurring *RAS* and *BRAF* mutations and how that might impact outcomes.

The remaining two long-term survivors both harbored the *KRAS* G12D mutation. One patient was receiving fourth-line treatment with TAS-102 and bevacizumab as of August 2023, while the other underwent right hemicolectomy and liver metastasectomy following conversion therapy with FOLFOX, followed by FOLFIRI and bevacizumab. The *KRAS* G12D mutation is among the most commonly identified *KRAS* alterations in colorectal and other gastrointestinal carcinomas (6, 24). Interestingly, this mutation has been associated with a more favorable OS compared to other *KRAS* variants in colorectal cancer (6). These observations raise the hypothesis that *KRAS* G12D may confer a prognostic advantage. Further investigation is warranted to explore the biological and clinical implications of this specific mutational profile.

The V600E mutation normally comprises approximately 90% of *BRAF* mutations present in CRC (23). Surprisingly, of the 13 patients in this series, only two possessed *BRAF* V600E mutation (15.4%). One of the two patients is still living as described above. The other, also female, had cecal primary with metastasis to liver and lung, concomitant *NRAS* Q61K mutation, unknown microsatellite status, and 9 muts/mB. She only lived four years from diagnosis despite multiple lines of treatment. This raises the question of whether non-V600E *BRAF* mutations may be more common in the presence of concomitant *RAS* expression.

Though still limited, there have been increasing amounts of data and studies describing concomitant *RAS* and *BRAF* mutations in mCRC. Despite this, it remains challenging to draw clear conclusions regarding the prognostic role of these concomitant mutations, their impact on PFS, OS, or how current chemotherapy regimens can be modified or combined to gain better control of the disease. The limitations of the current study include its small sample size, its retrospective nature, its lack of comparison group, and selection bias in that it represents a sample of patients from one small geographic area. Further, we are unable to draw conclusions about whether concomitant mutations effects OS as we did not compare our sample to those without concomitant mutations.

Our cohort suggests that double-mutated mCRC may have a metastatic pattern more similar to *RAS*-mutant CRC than solitary *BRAF*-mutated disease. It posits that *KRAS* G12D may contribute to improved prognosis even in the presence of *BRAF* mutation. And it questions the existing literature that the group I and II *BRAF* mutations are associated with worse prognosis. It also raises the question of whether other factors, like the presence of concomitant *RAS* family mutation can alter the trajectory of patients’ overall disease course. Ultimately, this study elucidates the need for more research on larger populations of patients who possess concomitant *RAS* and *BRAF* mutations in mCRC.

## Data Availability

The data analyzed in this study is subject to the following licenses/restrictions: Guardant360 and FoundationOne are Next-Generation Sequencing (NGS) Vendors. Wake Forest has established data use agreements with both Guardant360 and FoundationOne, allowing them to use the data for research purposes. Requests to access these datasets should be directed to https://guardanthealth.com/contact/; datacollaborations@foundationmedicine.com.
