# Supplementary material for: A compilation of 13 patients with metastatic colorectal cancer and concomitant BRAF and RAS family mutations
Source: Front Oncol. 2025 Aug 25;15:1621412. doi: 10.3389/fonc.2025.1621412 (PMC12414759; doi:10.3389/fonc.2025.1621412)
Supplement: Supplementary file 1 [file Table1.docx]

**FoundationOne CDx**

**Intended Use:** FoundationOne®CDx (F1CDx) is a next generation sequencing based in vitro diagnostic device for detection of substitutions, insertion and deletion alterations (indels), and copy number alterations (CNAs) in 324 genes and select gene rearrangements, as well as genomic signatures including microsatellite instability (MSI) and tumor mutational burden (TMB) using DNA isolated from formalin-fixed paraffin embedded (FFPE) tumor tissue specimens.

**Table 1.** Genes with full coding exonic regions included in FoundationOne®CDx for the detection of substitutions, insertions and deletions (indels), and copy number alterations (CNAs):


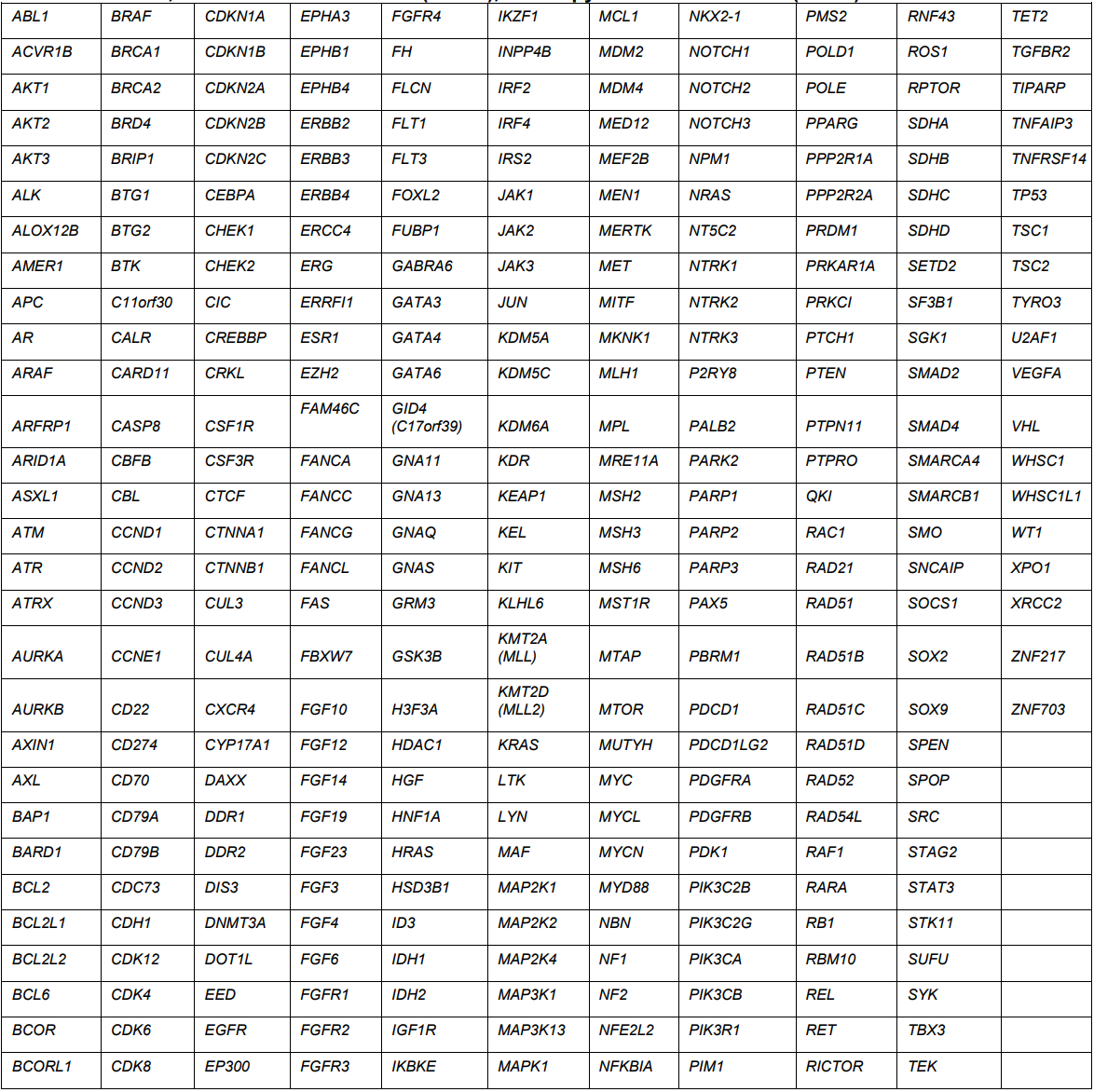


**For detailed description and to locate original source of material presented above:** [https://www.accessdata.fda.gov/cdrh_docs/pdf17/P170019S006C.pdf](https://urldefense.com/v3/__https:/www.accessdata.fda.gov/cdrh_docs/pdf17/P170019S006C.pdf__;!!Kv7QgGdTlhIaqSqT!MKLjDeg9fmiwLgch4W9CfOqpF4emt5yWQK-bM-8QHbrkP9ENrmBxeGIj8HCUdMufRR-Ky8tmYgY42n7FVlUgRjbDe3E$)
