# Supplementary material for: A compilation of 13 patients with metastatic colorectal cancer and concomitant BRAF and RAS family mutations
Source: Front Oncol. 2025 Aug 25;15:1621412. doi: 10.3389/fonc.2025.1621412 (PMC12414759; doi:10.3389/fonc.2025.1621412)
Supplement: Supplementary file 2 [file Table2.docx]

**Guardant360 CDx**

**Intended Use**: Guardant360® CDx is a qualitative next generation sequencing-based in vitro diagnostic device that uses targeted high throughput hybridization-based capture technology for detection of single nucleotide variants (SNVs), insertions and deletions (indels) in 55 genes, copy number amplifications (CNAs) in two (2) genes, and fusions in four (4) genes. Guardant360 CDx utilizes circulating cell-free DNA (cfDNA) from plasma of peripheral whole blood collected in Streck Cell-Free DNA Blood Collection Tubes (BCTs).

**Table 1**. Genes Containing Alterations Reported by Guardant360 CDx


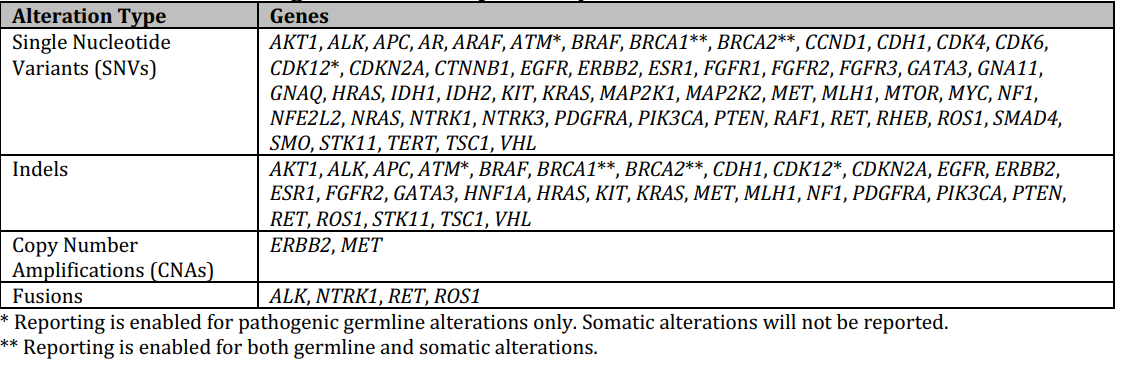


**For detailed description and to locate original source of material presented above:** <https://www.guardantcomplete.com/assets/pdf/Guardant360-CDx-Technical-Information-US.pdf>
